# Optimization of Ultrasound-Assisted Extraction and Structural Characterization of the Polysaccharide from Pumpkin (*Cucurbita moschata*) Seeds

**DOI:** 10.3390/molecules23051207

**Published:** 2018-05-18

**Authors:** Libo Wang, Long Cheng, Fangcheng Liu, Tengfei Li, Zeyuan Yu, Yaqin Xu, Yu Yang

**Affiliations:** 1 College of Science, Northeast Agricultural University, Harbin 150030, China; wanglibo@neau.edu.cn (L.W.); 13295617154@163.com (L.C.); 15645098212@163.com (F.L.); 18946196279@163.com (T.L.); xu-yaqin@163.com (Y.X.); yangyu_002@163.com (Y.Y.); 2 College of Horticulture, Northeast Agricultural University, Harbin 150030, China

**Keywords:** pumpkin seeds, polysaccharides, ultrasound-assisted extraction, response surface method, structural characterization

## Abstract

In the present study, ultrasound-assisted extraction (UAE) of crude polysaccharides (PSP) from pumpkin seeds was optimized by response surface method (RSM). The polysaccharide yield (2.29 ± 0.14%), which agreed closely with the theoretical predicted value 2.40%, was obtained under the optimal extraction conditions: extraction time 24 min, extraction temperature 50 °C, ultrasonic power 347 W, and liquid to solid ratio 23 mL/g. After further purification by two-step column chromatography, a novel polysaccharide (PSP-1) was isolated from pumpkin seeds. PSP-1 was composed of mannose, glucose, and galactose in a molar ratio of 1.00:4.26:5.78 with molecular weight of 3728 g/mol. 1D and 2D NMR spectroscopy analysis revealed that the backbone of PSP-1 was mainly formed by β→6)-β-d-Galp-(1→, →6)-α-d-Glcp-(1→, and →3,6)-β-d-Manp-(1→ with branching at *O*-3 and *O*-6 of →3,6)-β-d-Manp-(1→. Branch linkages were composed of α-d-Glcp-(1→ and →4)-α-d-Galp-(1→.

## 1. Introduction

Pumpkin seeds have been so commonly used in functional foods or medicines, which was ascribed to the valuable functional components [1]. Pumpkin seeds were rich in proteins, essential fatty acids, sterols, arginine, vitamins, and trace elements [2]. Previous studies showed that pumpkin seeds had health protective values, such as antioxidant, immunomodulatory, diuretic and anti-inflammatory activities [3,4]. Moreover, pumpkin seeds were also used to assist the healing process in wounds and hold back the infection of wounds in diabetic conditions [5].

Polysaccharides were polymeric carbohydrate structures which were made up of repeating units connected together through glycosidic bonds [6]. Over the years, a lot of researchers have reported that the polysaccharides extracted from natural source had immunomodulatory, hypolipidemic, antioxidant, anticancer, antitumor effects, and other biological activities [7,8,9]. Therefore, discovery and assessment of polysaccharides extracted from plants, microorganisms, and animals have become an area of research focus [9,10,11].

In recent years, ultrasound-assisted extraction (UAE) has been applied to effectively extract polysaccharides from different kinds of materials [12,13]. Compared with the conventional hot water extraction method, UAE can enhance the extraction yield, save operation time and streamline the operation process [14]. Response surface method (RSM) is a practical statistical method used for optimizing processing parameters [15]. The major advantage of RSM is that it can reduce the number of experimental groups and investigate the relation between the response and variables [16]. Therefore, RSM has been widely applied to accelerate and optimize the operation process for saving time, energy, and raw materials [17].

In our previous study, the parameters of hot water extraction were investigated by RSM, and the yield of crude polysaccharides from pumpkin seeds was 2.18%, which agreed well with the predicted value [18]. After rehydration with distilled water, the soluble crude polysaccharides were fractionated into three polysaccharides, named PSP-30, PSP-60, and PSP-80, by different gradient concentrations of ethanol (0–30%, 30–60%, 60–80%). Three polysaccharides were basically composed of the same monosaccharide composition and showed good antioxidant activity [19]. A homogeneous heteropolysaccharide, PSP-I, was obtained by further purification. PSP-I displayed certain antioxidant activities and a significant inhibitory effect on α-amylase [20]. Up to now, there was no study about UAE optimization of polysaccharides from pumpkin seeds. In addition, it was reported that the polysaccharides extracted with different methods showed different structure characterization [21].

In the present study, ultrasonic-assisted extraction of the polysaccharides from pumpkin seeds was investigated. The effects of four variables (extraction time, extraction temperature, ultrasonic power, and liquid to solid ratio) on the yield of PSP were optimized by RSM. After two-step column isolation from PSP, the purified fraction (PSP-1) was obtained and analyzed for detailed structure by FT-IR, periodate oxidation, Smith degradation, and NMR (^1^H, ^13^C, COSY, HSQC, and HMBC).

## 2. Results and Discussion

### 2.1. Single Factor Tests of UAE

#### 2.1.1. Effect of Different Extraction Time on the Yield of PSP

**Figure 1:**
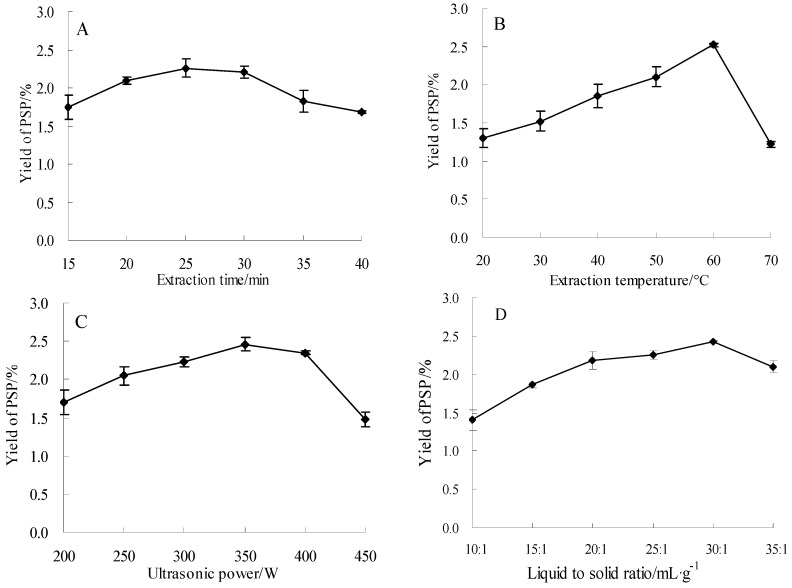
Effects of different time (**A**); temperature (**B**); power (**C**); and liquid to solid ratios (**D**) on the yield of polysaccharides (PSP).

In this experiment, the influence of different extraction time (15, 20, 25, 30, 35, and 40 min) on the yield of PSP was examined while keeping liquid to solid ratio at 15:1 mL/g, ultrasonic power at 300 W, and extraction temperature at 50 °C. As shown in Figure 1A, the yield of PSP achieved a maximum (2.26 ± 0.12%) when the extraction time was 25 min and decreased thereafter. The results indicated that longer extraction time can induce degradation of the polysaccharides and corresponding decrease in the yield of PSP [22]. Therefore, 25 min was taken as the optimal extraction time here.

#### 2.1.2. Effect of Different Extraction Temperature on the Yield of PSP

UAE process was executed at different temperature (20, 30, 40, 50, 60, and 70 °C) to analyze the influence of temperature on the yield of PSP. The extraction time, ultrasonic power, and liquid to solid ratio were set at 25 min, 300 W, and 15:1 mL/g, respectively. Figure 1B revealed the yield of PSP enhanced slowly with increasing temperature, and eventually reached a maximum at 60 °C, and the yield decreased when the temperature was over 60 °C. This might be attributed to the reason that the surface tension and the viscosity of solvent were reduced, and the steam pressure in small bubbles was increased at higher temperature, which will result in the decrease of the ultrasonic cavitation and the mass-transfer intensity [12]. Based on the results, 60 °C was used in further experiments.

#### 2.1.3. Effect of Different Ultrasonic Power on the Yield of PSP

UAE procedure was executed at different power (200, 250, 300, 350, 400, and 450 W) while setting the other factors as follows: extraction temperature of 60 °C, liquid to solid ratio of 15:1 mL/g, and extraction time of 25 min. Figure 1C revealed the yield of PSP increased with increasing ultrasonic power until value of 350 W, and after the point, it began to decrease. It has been reported that high-intensity ultrasonic wave can cause a large number of microscopic bubbles in the reaction, which might reduce the efficiency of ultrasonic energy transmitted into the medium and decreased the yield [13]. Therefore, 350 W was the optimal power here.

#### 2.1.4. Effect of Different Liquid to Solid Ratios on the Yield of PSP

In the present research, the influence of different liquid to solid ratios (10:1, 15:1, 20:1, 25:1, 30:1, and 35:1 mL/g) on the yield of PSP was investigated when other experimental conditions (power, temperature, and time) were fixed at 350 W, 60 °C, and 25 min, respectively.

The yield of PSP enhanced with increasing liquid to solid ratio, and attained the highest value when the ratio was 30:1 (Figure 1D). However, there was no increase when the ratio was higher than 30:1. This might be due to that the excessive liquid could decrease the ultrasonic energy attached to the unit volume, leading to decrease of extraction yield [23]. According to these results, a liquid to solid ratio of 30:1 mL/g was chosen for the next extraction process.

On the basis of these results, 25 min, 30:1 mL/g, 60 °C, and 350 W were employed for the succeeding RSM experiments.

### 2.2. Optimization of the Yield of PSP by RSM

#### 2.2.1. Statistical Analysis and Model Fitting

In the current Box-Behnken design (BBD), 29 runs were used to optimize the four individual variables. The coded values of the observed variables and the yields of PSP under different conditions were shown in Table 1, the yield values varied from 1.78% to 2.42%. After these data were handle with multiple regression analysis, a second-order polynomial equation for the yield of PSP could be represented by Equation (1):

*Y* = 2.38 − 0.073*X*_1_ − 0.048*X*_2_ − 0.032*X*_3_ − 0.061*X*_4_ − 0.058*X*_1_*X*_2_ − 0.0025*X*_1_*X*_3_ + 0.020*X*_1_*X*_4_ − 0.12*X*_2_*X*_3_ − 0.023*X*_2_*X*_4_ + 0.010*X*_3_*X*_4_ − 0.13*X*_1_^2^ − 0.19*X*_2_^2^ − 0.24*X*_3_^2^ − 0.10*X*_4_^2^ (1)

where *Y* was the predicted yield of PSP, *X*_1_, *X*_2_, *X*_3_, and *X*_4_ were the coded values for extraction time, extraction temperature, ultrasonic power, and liquid to solid ratio, respectively.

Analysis of variance (ANOVA) results, the adequacy of the regression model and fitness of the models obtained were listed in Table 2. *F*-value (14.24) and *p*-value (*p* < 0.0001) revealed the model was very significant. Meanwhile, the lack-of-fit *F*-value of 3.79 also indicated the model was significant.

The determination coefficient *R*^2^ was 0.9344, which indicated that the model was suitable for prediction in the scope of experimental variables. The adjusted determination coefficient (*R*^2^_adj_ = 0.8688) further proved that the experimental values were quite consistent with the predicted values. In addition, the coefficient of variation value (CV = 2.93%) obviously indicated that the experimental values were sure and accurate [16].

Table 2 showed that the linear coefficients (*X*_1_, *X*_2_, *X*_4_), the quadratic term coefficients (*X*_1_^2^, *X*_2_^2^, *X*_3_^2^, *X*_4_^2^), and the interaction between time and temperature (*X*_2_*X*_3_) were all significant at the level of *P* < 0.05 or *P* < 0.01. The other term coefficients were not significant (*p* > 0.05). Hence, *X*_1_, *X*_2_, *X*_3_, *X*_4_, *X*_1_^2^, *X*_2_^2^, *X*_3_^2^, *X*_4_^2^, and *X*_2_*X*_3_ were crucial factors in the extraction process of PSP. After all the insignificant factors were removed, the final empirical model could be described by the following equation.

*Y* = 2.38 − 0.073*X*_1_ − 0.048*X*_2_ − 0.061*X*_4_ − 0.12*X*_2_*X*_3_ − 0.13*X*_1_^2^ − 0.19*X*_2_^2^ − 0.24*X*_3_^2^ − 0.10*X*_4_^2^ (2)

#### 2.2.2. Diagnostics of Model Adequacy

**Figure 2:**
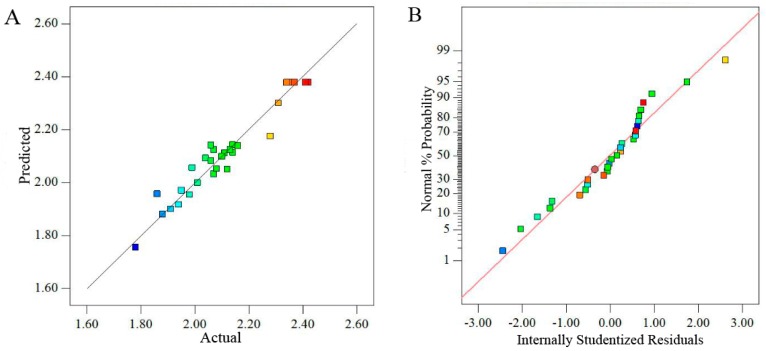

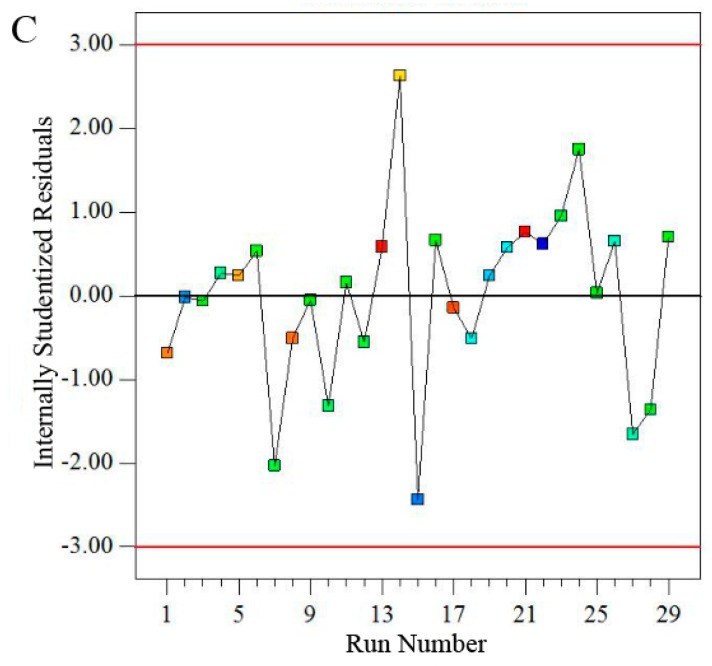
Diagnostic plots for the adequacy of proposed model. ((**A**) Plot of predicted and actual values; (**B**) the normal % probability plot; (**C**) plot of internally studentized residuals versus actual runs).

Figure 2A showed that the predicted values obtained from the model were in agreement with the experimental data. At the same time, the normal probability plots of residuals did not indicate evidence of serious deviation from normality in Figure 2B. In addition, the residuals were scattered randomly around ±3.00 (Figure 2C), which demonstrated all the data points lay within the acceptable limits.

#### 2.2.3. Analysis of Response Surfaces

**Figure 3:**
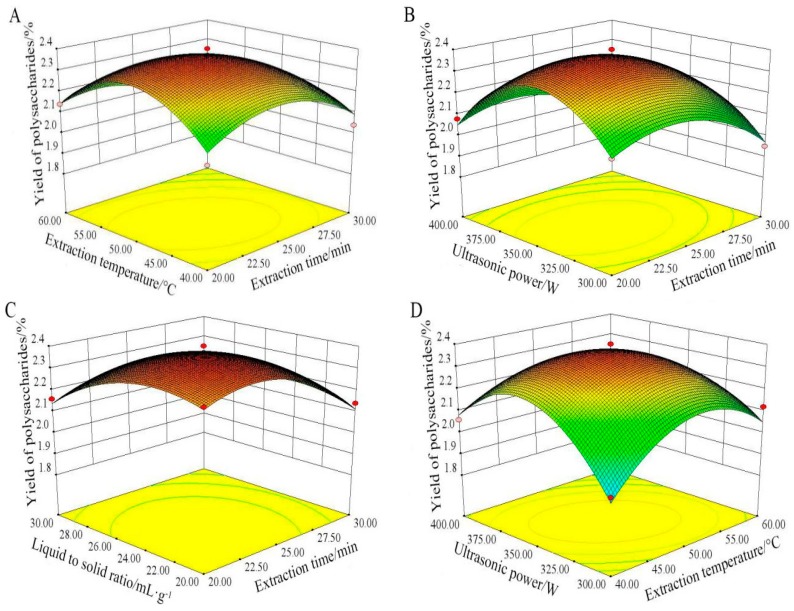
Tri-dimensional response surface plots showing the interactions between variables on PSP extraction. ((**A**) Extraction temperature and extraction time; (**B**) ultrasonic power and extraction time; (**C**) liquid to solid ratio and extraction time; (**D**) ultrasonic power and extraction temperature).

As shown in Figure 3, the three-dimensional response surface were very helpful to predict the type of the interaction between the two measured variables, the relationship between responses and experimental levels of each variable, and to locate the optimum conditions for maximum yield of PSP.

Figure 3A showed the interaction between extraction time and extraction temperature when ultrasonic power and liquid to solid ratio were fixed at 350 W and 25:1 mL/g, respectively. The yield of PSP increased at first, and then decreased with the two variables increasing thereafter. The highest yield should be found with the extraction time range of 20–28 min and the extraction temperature range of 40–55 °C, respectively.

Figure 3B revealed the effect of ultrasonic power and extraction time for the polysaccharides yield when other two factors were kept at center point. The yield of PSP initially increased when ultrasonic power was maintained at a lower level, and then decreased. A great increase of PSP yield was obtained when the ultrasonic power increased in the range from 300 to 375 W.

Figure 3C showed the interaction of extraction time and liquid to solid ratio on the polysaccharides yield at fixed extraction temperature (50 °C) and ultrasonic power (350 W). The maximum yield of PSP could be obtained when extraction time and liquid to solid ratio were kept at 27 min and 26:1, respectively.

As expected, the yield of PSP increased significantly with temperature changed from 40 to 55 °C (Figure 3D). Likewise, the yield of PSP increased at first, and then decreased when the ultrasonic power increased in the range from 300 to 375 W. Therefore, the maximum yield of PSP could be achieved at 55 °C and 375 W.

The above data indicated the interaction of ultrasonic power and extraction temperature caused significant effects for the extraction yield of PSP (*p* < 0.01), and the reciprocal interactions between other variables (temperature and time, ultrasonic power and time, liquid to solid ratio and time) were insignificant (*p* > 0.05) in Table 3. This result was in accord with previous studies [24,25], which also showed that the interactions between ultrasonic power and extraction time, as well as ultrasonic power and liquid to solid ratio, were not significant.

By analyzing these three-dimensional plots in Figure 3, the optimum levels of the variables were as follows: ultrasonic power 346.89 W, extraction temperature 49.56 °C, extraction time 23.52 min, and liquid to solid ratio 23.37 mL/g. According to the optimum conditions, the predicted value of the yield was 2.40%.

#### 2.2.4. Validation of the Predictive Models

In order to provide convenient operation, the optimal parameters were fitted as follows: extraction time 24 min, extraction temperature 50 °C, ultrasonic power 347 W, and liquid to solid ratio 23 mL/g. According to the optimal conditions, the experimental value of response was 2.29 ± 0.12%, which agreed nearly with the predicted value (2.40%). These data showed the model was satisfactory and precise for the prediction of PSP extraction.

According to our previous report [18], the optimal hot water extraction conditions for pumpkin seeds polysaccharides were as follows: extraction time 2.5 h, extraction temperature 60 °C, and liquid to solid ratio 40:1 mL/g. The yield of crude polysaccharides from pumpkin seeds was 2.18%, obviously, UAE exhibited excellent efficiency with higher yield, shorter time, and lower solvent consumption.

UAE has been widely employed for the extraction of polysaccharides from various sources, such as *Lycium barbarum*, *Tremella mesenterica*, *Trametes orientalis*, dried longan pulp, and annatto seeds [12,15,22,26,27]. All these results preliminarily showed UAE was a more rapid and efficient technique than other conventional extractions, and provided a scientific basis for the further use of the UAE in food industry.

### 2.3. Purification and Preliminary Characterisation of PSP-1

**Figure 4:**
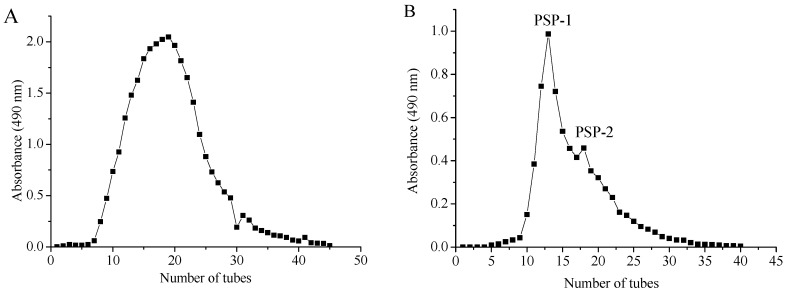

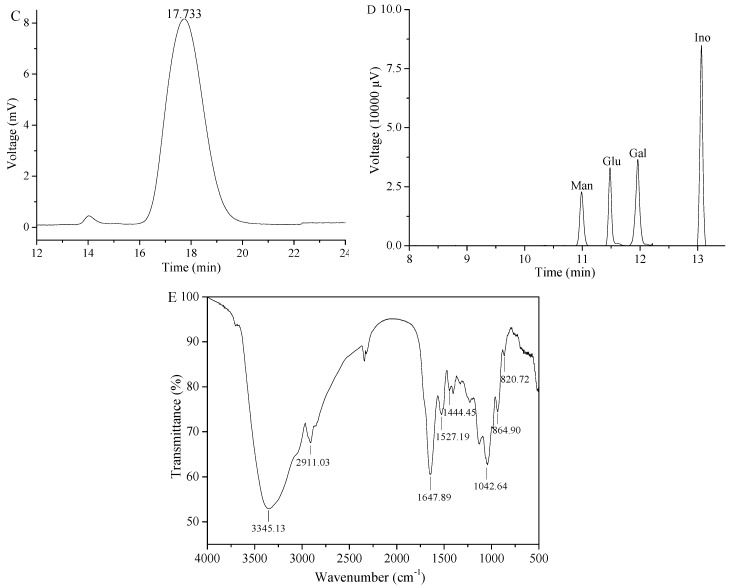
Elution profiles and characterization of polysaccharides. ((**A**) PSP on DEAE cellulose DE-23 column; (**B**) Further elution of PSP on Sephadex G-25; (**C**) HPLC elution curve of PSP-1; (**D**) GC profile of PSP-1 with acid hydrolysis and acetylation; (**E**) FT-IR spectrum of PSP-1).

PSP-1 was obtained through further purification of PSP by using ion-exchange chromatography of diethylaminoethyl (DEAE) cellulose DE-23 column and gel-filtration chromatography of Sephadex G-25 (shown in Figure 4A,B). The fraction PSP-2 was conjugate with PSP-1 and much lower than that of PSP-1, therefore, the top peak of PSP-1 with a yield of 16.65% based on the dried crude polysaccharides was collected. The purity of PSP-1 was determined to be 86.43% with a protein content of 7.85%, and there was no uronic acid in PSP-1. As shown in Figure 4C, PSP-1 exhibited a single and symmetric peak in the HPGPC chromatogram, indicating that PSP-1 was a homogeneous polysaccharide. According to the regression equation (lg*M*_w_ = −0.358*t* + 9.92, *R*^2^ = 0.9985), the *M*_w_ of PSP-1 was calculated as 3728 g/mol with the retention time of 17.733 min. The monosaccharide composition of PSP-1 was analyzed by comparing the retention time against standards (Figure 4D). GC analysis demonstrated that PSP-1 was composed of mannose, glucose, and galactose in a molar ratio of 1.00:4.26:5.78, respectively.

In our previous study, the crude polysaccharides from pumpkin seeds were obtained by hot water extraction, then PSP-I with an average *M*_w_ of 21,083 g/mol was isolated after two-step column purification [20]. Compared with PSP-I, the *M*_w_ of PSP-1 was smaller. It has been reported that the polysaccharides in the rhizoma of *Dioscorea hemsleyi* were extracted using UAE, cold water extraction, warm water extraction, and hot water extraction, respectively, the molecular weight of polysaccharides obtained by UAE was the smallest among four methods [21]. Moreover, some researches showed that the high-molecular-weight polysaccharides were degraded into the low-molecular-weight polysaccharides after ultrasonic treatment, but the main structure of the polysaccharides was not changed [26,27]. All these data indicated UAE was more suitable for preparing the polysaccharides with lower *M*_w_.

### 2.4. FT-IR Spectrum Analysis of PSP-1

The FT-IR spectrum of PSP-1 is shown in Figure 4E, and it was obvious that PSP-1 possessed typical absorptive peaks for glycosidic structures. The strong absorptive peak at 3345 cm^−1^ showed that there were intermolecular and intramolecular hydrogen bonds. The characteristic absorption bands at 2911 and 1444 cm^−1^ were associated with the C–H stretching vibration and C–H bending vibration, respectively. The spectrum also showed C=O stretching vibration at 1647 cm^−1^ and N–H variable angle vibration stretching at 1527 cm^−1^ [28]. In addition, the absorption peaks around 1150 to 1050 cm^−1^ were ascribed to C–O–C and C–O–H vibration, indicating the existence of pyranose [29]. The characteristic absorption bands at 820 and 864 cm^−1^ suggested that both α- and β-configurations of sugar units existed in PSP-1 [30].

### 2.5. Periodate Oxidation and Smith Degradation Analysis

The results of periodate oxidation demonstrated that the consumption of NaIO_4_ was 0.0378 mol, and the production of formic acid was 0.0144 mol per mole of sugar residue, indicating that the presence of 1→linked or (1→6)-linked monosaccharides in PSP-1. The amount of consumed periodate was more than two times the amount of generated formic acid (>0.0144 mol × 2), which showed the existence of 1→2 or (1→4)-linked sugar residues. After Smith degradation, the existence of erythritol and glycerol demonstrated that (1→6)-linked and (1→4)-linked glycosyl bonds were presented in PSP-1. The absence of glucose indicated that all Glc linkages could be oxidized by periodate, namely 1→linkage, (1→6)-linkage, (1→2)-linkage and (1→2,6)-linkage. The detailed structure determination of PSP-1 would be confirmed systematically with NMR analysis.

### 2.6. Analysis of NMR

**Figure 5:**
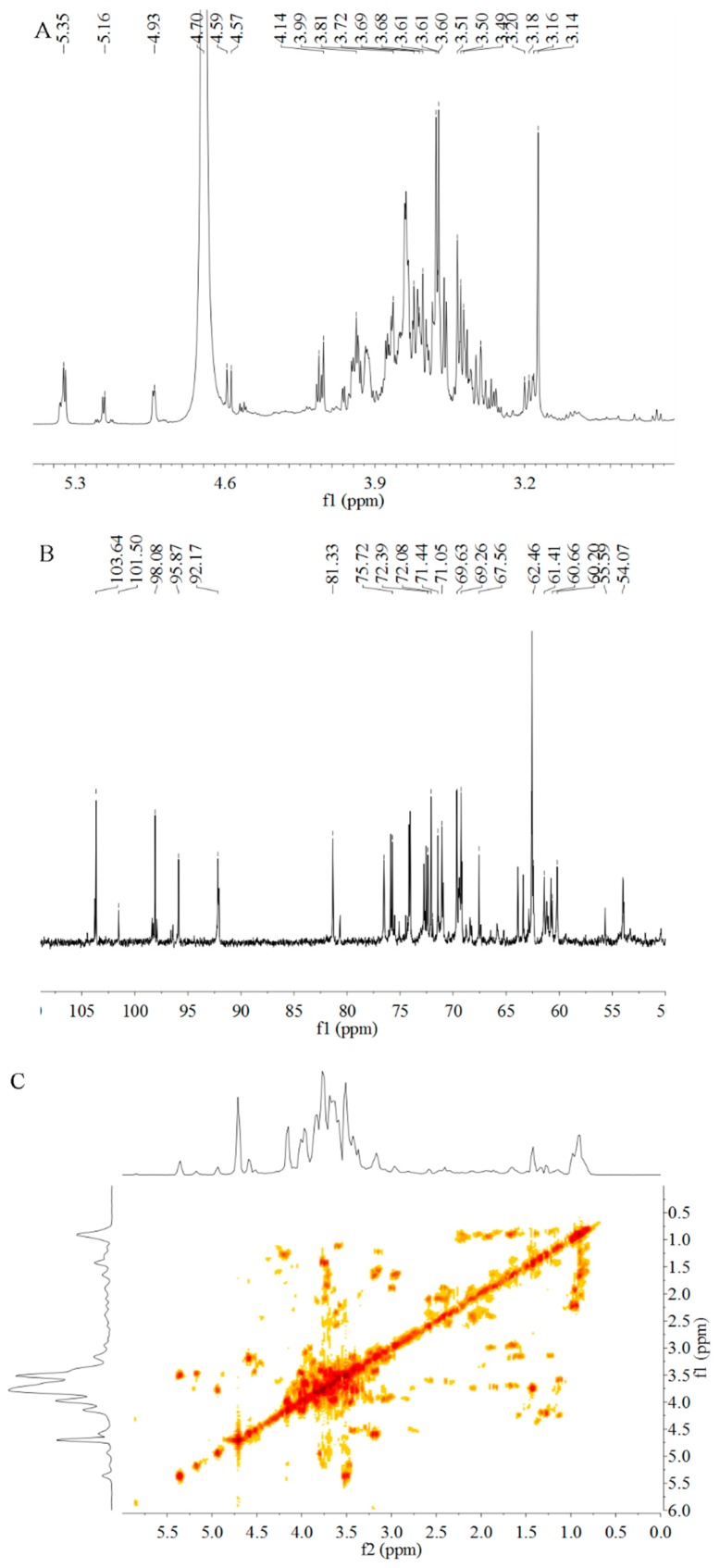

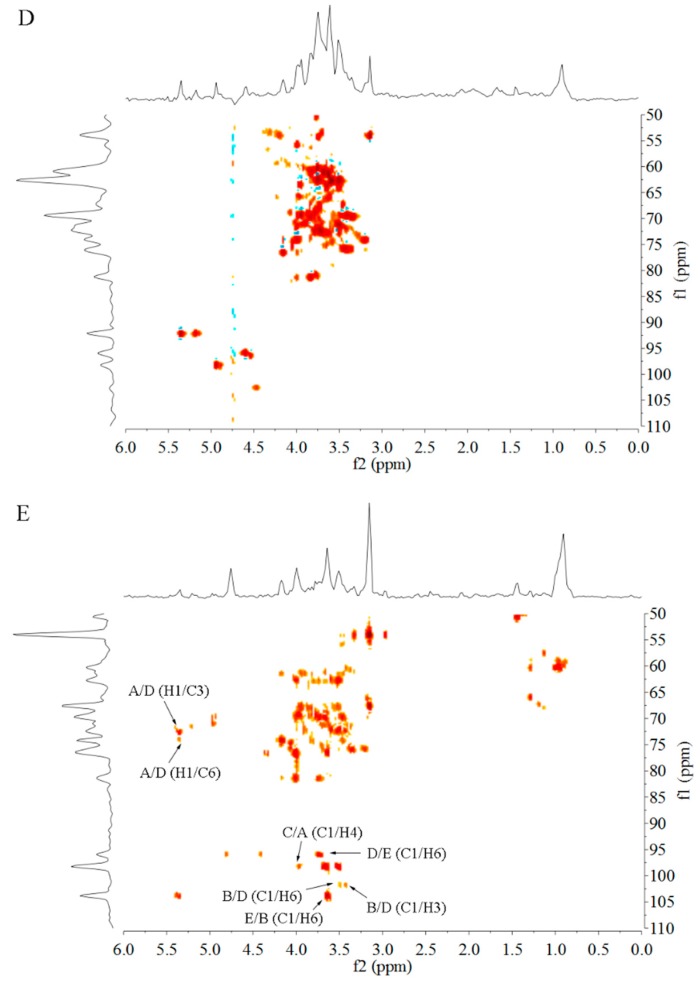
NMR spectra of PSP-1. (**A**) ^1^H NMR; (**B**) ^13^C NMR; (**C**) ^1^H–^1^H COSY; (**D**) ^1^H–^13^C HSQC; and (**E**) ^1^H–^13^C HMBC.

1D and 2D NMR spectra of PSP-1 were showed in Figure 5. The signals showed typical distribution of polysaccharides ranging from δ_H_ 3.0–5.5 ppm (in Figure 5A) and δ_C_ 60–110 ppm (in Figure 5B) [31,32]. The anomeric proton signals at δ 5.35, 5.16, 4.93, 4.59, and 4.57 ppm, as well as the anomeric carbon signals at δ 92.17, 95.87, 98.08, 101.50, and 103.64 ppm, revealed that PSP-1 contains five types of monosaccharide residues. At the same time, there were no signals between δ 107–109 ppm (C-1) and δ 82–84 ppm (C3–C5) of furan ring in ^13^C NMR spectrum, which indicated that all sugar residues were all in the pyranose form [33], and this was consistent with the results of FT-IR data.

All the proton chemical shifts (H-1 to H-6) were identified from COSY spectrum (Figure 5C), and the chemical shifts of the C-1 to C-6 were assigned from the HSQC spectrum (Figure 5D). The glycosidic linkages between sugar residues were determined by HMBC spectrum (Figure 5E). Combined with periodate oxidation results, Smith degradation analysis, 2D NMR spectra, and documented data, ^1^H and ^13^C NMR chemical shifts of the main residues from PSP-1 were shown in Table 3. The anomeric proton and carbon signals at 5.35/92.17, 5.16/101.50, 4.93/98.08, 4.59/95.87, 4.57/103.64 were assigned to the H-1/C-1 of A: →4)-α-d-Galp-(1→, B: →6)-α-d-Glcp-(1→, C: α-d-Glcp-(1→, D: →3,6)-β-d-Manp-(1→, and E: →6)-β-d-Galp-(1→ residues, respectively [34,35,36,37,38]. Signals at δ 3.65/75.13, 3.36/69.65, 3.84/81.31, 3.41/68.02, and 3.61/62.63 ppm were attributed to C-2/H-2–C-6/H-6 of →4)-α-d-Galp-(1→, respectively [39]. Signals at δ 3.99/72.79, 3.90/76.51, 3.52/71.38, 4.06/76.35, and 3.62/65.94 were attributed to C-2/H-2–C-6/H-6 of →6)-α-d-Glcp-(1→ [35], respectively. Signals at δ 3.99/72.99, 3.90/76.51, 3.95/69.26, 3.82/72.96, and 3.61/62.63 were attributed to C-2/H-2–C-6/H-6 of α-d-Glcp-(1→, respectively [36]. Signals at δ 3.81/66.29, 3.50/72.70, 3.54/72.20, 71.66/3.60, and 3.43/75.82 were attributed to C-2/H-2–C-6/H-6 of →3,6)-β-d-Manp-(1→ [37], respectively. Moreover, δ 3.44/68.02 (H-2/C-2), δ 3.83/73.23 (H-3/C-3), δ 4.02/70.72 (H-4/C-4), δ 3.96/74.22 (H-5/C-5) and δ 3.73/69.66 (H-6/C-6) were assigned to →6)-β-d-Galp-(1→ [38,40].

**Figure 6:**
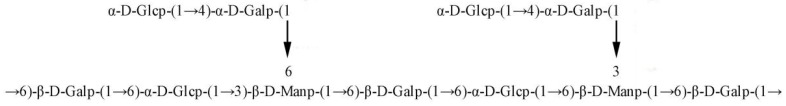
The backbone of PSP-1.

As shown in Figure 5E, seven main inter-residual cross peaks were observed, including A H-1 (5.35 ppm) to D C-3 (72.20 ppm), A H-1 (5.35) to D C-6 (75.82), B C-1 (101.50 ppm) to D H-6 (3.43 ppm), B C-1 (101.50 ppm) to D H-3 (3.50 ppm), C C-1 (98.08 ppm) to A H-4 (3.84 ppm), D C-1 (95.87 ppm) to E H-4 (3.20 ppm) and E C-1 (103.64 ppm) to B H-6 (3.62 ppm). Based on all these lines of evidence, the probable structure of PSP-1 was designated in Figure 6.

### 2.7. SEM Analysis

**Figure 7:**
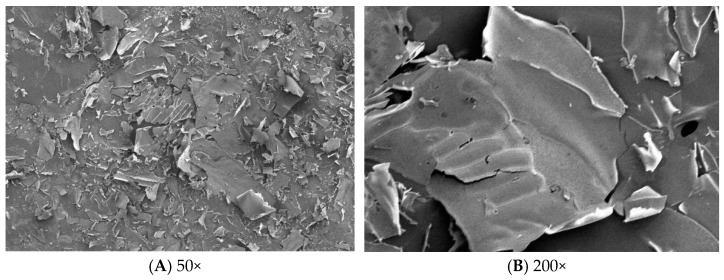
SEM images.

SEM can be used to analyze the surface morphology of polysaccharides. The microstructures of PSP-1 at ×50 times and ×200 times were shown in Figure 7. PSP-1 exhibited flake-like structure and rough surface.

## 3. Materials and Methods

### 3.1. Materials

Naked pumpkin seeds were obtained from Baoku Seed Research Institute (Nehe, Heilongjiang, China). Before extraction, the seeds were dried in a 202-2A electrothermal constant-temperature dry box (Tianjin Taisite Instrument Co., Ltd., Tianjin, China) at 60 °C, ground into powder in a FW100 universal high-speed smashing machine (Tianjin Taisite Instrument Co., Ltd., China), and screened through a 120 mesh sieve. Dry powder of pumpkin seeds was defatted with anhydrous ether. The resulting powder was stored for further polysaccharides extraction. Dextrans of different molecular weights (T-10, T-40, T-70, T-110, and T-500) were provided by Baierdi Biotechnology Co. (Beijing, China). DEAE cellulose DE-23 and Sephadex G-25 were purchased from Whatman Ltd. (Shanghai, China). Acetic anhydride and pyridine were obtained from Tianjin Regent Chemicals Ltd. (Tianjin, China). All the other chemicals used in present experiment were of analytical grade.

### 3.2. Extraction of PSP

The pumpkin seed powder (3.0 g) was mixed with deionized water under different liquid to solid ratios (10:1–35:1 mL/g) in a 250 mL beaker, then the polysaccharides were extracted at a designed temperatures (20–70 °C) in an ultrasonic cell disintegrator (JY92-2D, Ningbo Xinzhi Biological Technology Co., Ltd., Ningbo, China) at various ultrasonic power (200–450 W) for various periods (15–40 min). Then the extracts were centrifuged at 3500 rpm (20NKIA SC-3610, Anhui Zhongke Zhongjia Science Instrument Co., Ltd., Hefei, Anhui, China) for 10 min and the supernatant was concentrated by vacuum rotary evaporator below 50 °C. The concentrate was precipitated at 4 °C for 12 h with 80% (*v*/*v*) ethanol. The sediment was gathered by centrifugation (3500 rpm for 10 min), and washed with anhydrous ethanol and acetone, then lyophilized to obtain the polysaccharides (PSP).

The phenol–sulfuric acid method was applied to evaluate the content of total polysaccharides with d-glucose as a criterion [20]. The yield of polysaccharides *Y* (%) was calculated according to the following formula:

*Y*(%) = (*a* × *v*)/*b* ×100% (3)

where *a* is the concentration of the polysaccharides in the sample solution (mg/mL), *v* is the volume of the sample solution (mL), and *b* is the dried sample mass (mg).

### 3.3. Single Factor Experiments of UAE

The preliminary ranges of extraction time (15, 20, 25, 30, 35, and 40 min), extraction temperature (20, 30, 40, 50, 60, and 70 °C), ultrasonic power (200, 250, 300, 350, 400, and 450 W), and liquid to solid ratio (10:1, 15:1, 20:1, 25:1, 30:1, and 35:1 mL/g) were determined via single factor experiment, namely, one factor was altered at a designed range, but the other factors remained the same in all experiments. The effect degree of each factor was assessed by the yield of PSP.

### 3.4. Experimental Design of RSM

#### 3.4.1. Optimization of UAE by RSM

Based on single factor experiment, a BBD with four-variable and three-level was employed to statistically optimize the operation process. Extraction time (*X*_1_), extraction temperature (*X*_2_), ultrasonic power (*X*_3_), and liquid to solid ratio (*X*_4_) were selected for independent variables to be optimized for the extraction of PSP. The yield of PSP (*Y*) was regarded as responses. Table 4 showed the coded levels of four factors.

The four factors were coded based on the Equation (4):

*X_i_* = (*x*_i_ − *x*_0_)/Δ*x* i *=* 1, 2, 3, 4 (4)

where *X*_i_ is the (dimensionless) coded value of a factor, *x*_i_ is the real value of a factor, *x*_0_ was the real value of *x*_i_ at the center point, and Δ*x* was the step change value.

The regression model proposed for response was given below:

(5) $$Y=\beta_{0}+\sum_{i=1}^{4}\beta_{i}X_{i}+\sum_{i=1}^{4}\beta_{\mathrm{ii}}X_{i}{}^{2}+\sum_{i=1}^{3}\sum_{j=i+1}^{4}\beta_{\mathrm{ij}}X_{i}X_{j}$$

where *Y* is the dependent variable; *β*_0,_ *β*_i_, *β*_ii_, and *β*_ij_ are the regression coefficients for intercept, linearity, square, and interaction, respectively; *X*_i_ and *X*_j_ are input variables that influence the response variable *Y*.

#### 3.4.2. Verification of Predictive Model

To verify the availability of the mathematical model, three parallel experiments were conducted under the optimized conditions. Moreover, the reasonability of the mathematical model was based on detailed comparisons between the means of the experiments and predicted value of the developed model.

### 3.5. Purification and Isolation of PSP-1

Crude polysaccharides were dissolved in water and separated by a DEAE cellulose DE-23 column (3.0 cm × 60 cm). The column was eluted with distilled water at a flow rate of 1.0 mL/min and one main elution peak was obtained by phenol–sulfuric acid method at 490 nm. Then, a Sephadex G-25 column (1.8 cm × 50 cm) was used to further purification with distilled water as eluant at a flow rate of 1.0 mL/min. The eluate (5 mL/tube) was collected by phenol-sulfuric acid method. Two conjugate fractions, coded PSP-1 and PSP-2, were obtained. The top peak of PSP-1 fraction was collected for structure characterization. The protein content of PSP-1 was determined by Bradford method [41], and the content of uronic acid was determined according to carbazole–sulfuric acid method by using d-galacturonic acid as the standard [42].

### 3.6. Structural Characterization of PSP-1

#### 3.6.1. Determination of Molecular Weight

The molecular weight (*M*_w_) of PSP-1 was measured by using high performance liquid chromatography (HPLC, LC-10AVP, Shimadzu Corporation, Kyoto, Japan), equipped with a Waters Ultrahydrogel 2000 column (7.8 mm × 30 cm) and a model 2410 refractive index detector (RID). The sample solution (2.0 mg/mL) was injected into the detector after passing through 0.45 μm filter. The deionized water was regarded as flow phase, and the elution rate was 0.8 mL/min. The standard curve, the elution time plotted against the logarithm of molecular weight, was made using dextran T standards (T-10, T-40, T-70, T-110, and T-500).

#### 3.6.2. Analysis of Monosaccharide Composition

Gas chromatography (GC, GC-2010, Shimadzu Corporation, Kyoto, Japan) equipped with an RTX-1701 silica capillary column (30.0 m × 0.25 mm × 0.25 µm), and a FID was used to analyze the monosaccharide components. The procedures were carried out according to the published paper [29].

#### 3.6.3. Infrared Analysis

PSP-1 and KBr powder were mixed and then pressed into 1 mm pellets for Fourier transform infrared (FT-IR) (ALPHA-T, BRUKER Co., DE, Billerica, MA, USA) measurement within the limits of mid-infrared region (4000–400 cm^−1^).

#### 3.6.4. Periodate Oxidation and Smith Degradation

The periodate oxidation and Smith degradation were performed based on the literature methods [43]. PSP-1 (50 mg) was added into 100 mL 0.015 mol/L NaIO_4_, and kept in the dark. The solution (0.2 mL) was taken out every 6 h and monitored at 223 nm until the value became stable. Then, 2.0 mL of reaction liquid was drawn out and titrated with 0.008 mol/L NaOH, to measure the production of formic acid. The rest of the reaction solution was dialyzed in distilled water for 48 h. Non-dialysates were reduced with excess NaBH_4_ at 25 °C for 12 h. The solution was concentrated and hydrolyzed with 2.0 mol/L TFA, acetylated, and analyzed by GC according to the method in Section 3.6.2.

#### 3.6.5. Nuclear Magnetic Resonance (NMR) Spectroscopy

PSP-1 (40 mg) was dissolved in D_2_O. The ^1^H and ^13^C NMR spectra were recorded on a Bruker AV-400 spectrometer (Bruker Corporation, Zurich, Switzerland). Two-dimensional spectra (COSY, HSQC, and HMBC) were collected with standard Bruker procedures.

#### 3.6.6. SEM Analysis

The polysaccharide PSP-1 were coated with a thin layer of gold under reduced pressure, and then examined using a JSM-6480A scanning electron microscope (SEM, JEOL, Ltd., Tokyo, Japan).

### 3.7. Statistical Analysis

Each experiment was carried out by three separate experiments, and data were showed as mean ± standard deviation. Statistical analysis was implemented with Design-Expert (8.0.6 Statease Inc., Minneapolis, MN, USA). *p* < 0.05 was defined as a statistically significant difference.

## 4. Conclusions

In the present study, the optimum UAE conditions were determined, and compared to hot water extraction, the yield of crude polysaccharides from pumpkin seeds was improved, with shorter time and lower solvent consumption. After column purification, a novel heteropolysaccharide PSP-1 with *M*_w_ of 3728 g/mol was isolated. 2D NMR spectroscopy confirmed that PSP-1 contained five sugar residues, and the primary structure was provided. The *M*_w_ of PSP-1 was relatively smaller than that of PSP-I, a polysaccharide extracted using hot water from pumpkin seeds. This result revealed that UAE was much more efficient and suitable for preparation of polysaccharides with lower molecular weight.

## Figures and Tables

**Table 1:** BBD and the results (means of triplicate tests) for the yield of PSP.

Number	*X*_1_ (Extraction Time, min)	*X*_2_ (Extraction Temperature, °C)	*X*_3_ (Ultrasonic Power, W)	*X*_4_ (Liquid to Solid Ratio, mL/g)	Yield of PSP (%)
1	0	1	0	−1	2.13
2	1	0	0	−1	2.14
3	0	0	−1	1	2.01
4	1	−1	0	0	2.04
5	0	0	0	0	2.34
6	0	0	0	0	2.35
7	0	1	0	1	1.86
8	0	−1	0	−1	2.28
9	−1	1	0	0	2.14
10	0	0	0	0	2.41
11	0	0	1	1	1.98
12	1	1	0	0	1.88
13	0	0	−1	−1	2.06
14	1	0	−1	0	1.95
15	0	−1	−1	0	1.94
16	0	1	1	0	1.78
17	1	0	0	1	2.07
18	0	−1	0	1	2.10
19	−1	0	−1	0	2.11
20	0	0	0	0	2.37
21	−1	0	1	0	2.08
22	0	1	−1	0	2.12
23	0	0	0	0	2.42
24	1	0	1	0	1.91
25	0	0	1	−1	1.99
26	−1	−1	0	0	2.07
27	0	−1	1	0	2.06
28	−1	0	0	1	2.16
29	−1	0	0	−1	2.31

**Table 2:** ANOVA for the regression model.

Source	Sum of Squares	DF	Mean Square	*F*-Value	*p*-Value	Significant
Model	0.76	14	0.054	14.24	<0.0001	**
*X*_1_	0.065	1	0.065	16.96	0.0010	**
*X*_2_	0.028	1	0.028	7.37	0.0168	*
*X*_3_	0.013	1	0.013	3.33	0.0893	
*X*_4_	0.044	1	0.044	11.67	0.0042	**
*X*_1_ *X*_2_	0.013	1	0.013	3.48	0.0833	
*X*_1_ *X*_3_	2.500 × 10^−5^	1	2.500 × 10^−5^	6.57 × 10^−3^	0.9365	
*X*_1_ *X*_4_	1.600 × 10^−3^	1	0.0016	0.42	0.5271	
*X*_2_ *X*_3_	0.053	1	0.053	13.91	0.0022	**
*X*_2_ *X*_4_	2.025 × 10^−3^	1	2.025 × 10^−3^	0.53	0.4777	
*X*_3_ *X*_4_	4.000 × 10^−4^	1	4.000 × 10^−3^	0.11	0.7505	
*X*_1_^2^	0.11	1	0.11	28.93	<0.0001	**
*X*_2_^2^	0.23	1	0.23	60.11	<0.0001	**
*X*_3_^2^	0.37	1	0.37	97.40	<0.0001	**
*X*_4_^2^	0.067	1	0.067	17.57	0.0009	**
Residual	0.053	14	3.804 × 10^−3^			
Lack of fit	0.048	10	4.818 × 10^−3^	3.79	0.1053	
Pure error	5.080 × 10^−3^	4	1.270 × 10^−3^			
Cor total	0.81	28				
CV%			2.93%			
*R* ^2^			0.9344			
adj-*R*^2^			0.8688			

Notes: ** *p* < 0.01 extremely significant; * *p* < 0.05 significant.

**Table 3:** ^1^H and ^13^C NMR chemical shifts of the main residues from PSP-1 in D_2_O.

Sugar Residues	Chemical Shifts, δ ^1^H/^13^C (ppm)					
	1	2	3	4	5	6
A →4)-α-d-Galp-(1→	5.35	3.65	3.36	3.84	3.41	3.61
	92.17	75.13	69.65	81.31	68.02	62.63
B →6)-α-d-Glcp-(1→	5.16	3.99	3.90	3.52	4.06	3.62
	101.50	72.79	76.51	71.38	76.35	65.94
C α-d-Glcp-(1→	4.93	3.99	3.90	3.95	3.82	3.61
	98.08	72.99	76.51	69.26	72.96	62.63
D →3,6)-β-d-Manp-(1→	4.59	3.81	3.50	3.54	3.60	3.43
	95.87	66.29	72.70	72.20	71.66	75.82
E →6)-β-d-Galp-(1→	4.57	3.44	3.83	4.02	3.96	3.73
	103.64	68.02	73.23	70.72	74.22	69.66

**Table 4:** Variable levels used in RSM.

Variables	Coded Variable Levels		
	−1	0	+1
*X*_1_ Extraction time/min	20	25	30
*X*_2_ Extraction temperature/°C	40	50	60
*X*_3_ Ultrasonic power/W	300	350	400
*X*_4_ Liquid to solid ratio/mL·g^−1^	20:1	25:1	30:1
